# Supplementary figures and images for: Loss of ninein interferes with osteoclast formation and causes premature ossification
Source: eLife. 2024 Jun 5;13:e93457. doi: 10.7554/eLife.93457 (PMC11175614; doi:10.7554/eLife.93457)

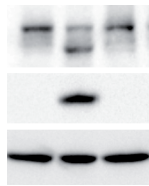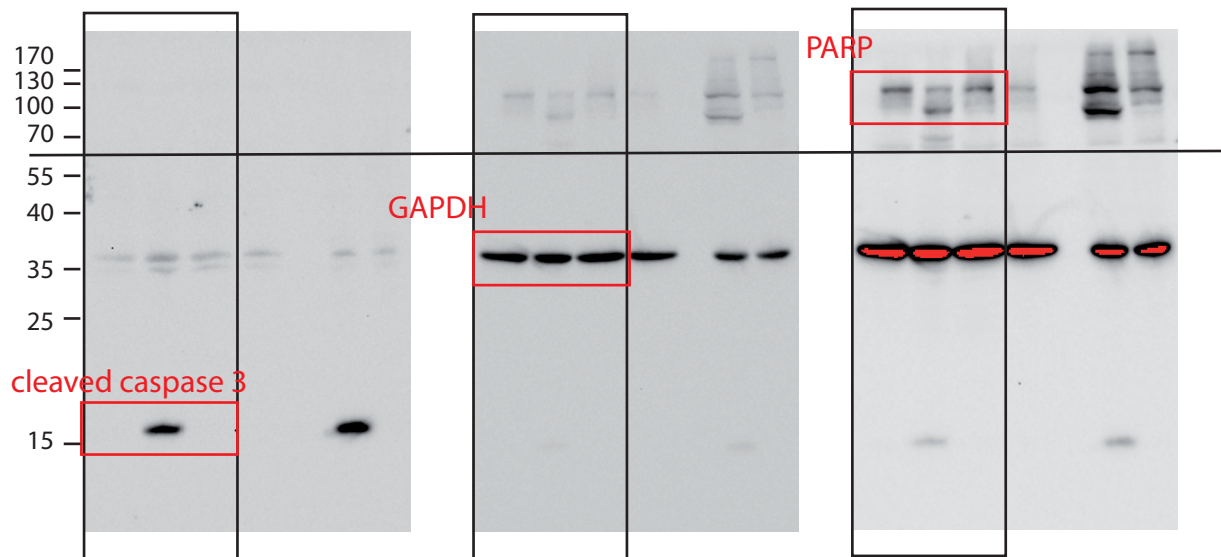

Supplement: Figure 7—figure supplement 1—source data 1. [file elife-93457-fig7-figsupp1-data1.pdf]
