# Supplementary material for: Loss of ninein interferes with osteoclast formation and causes premature ossification
Source: eLife. 2024 Jun 5;13:e93457. doi: 10.7554/eLife.93457 (PMC11175614; doi:10.7554/eLife.93457)
Supplement: Supplementary file 1. — Table, indicating the lengths of various bones from embryos at E16.5 and E18.5, from control and ninein del/del embryos. [file elife-93457-supp1.docx]

**Supplementary file 1: characteristics of forelimb and hindlimb bones in control and ninein-deleted mice.**

|  | Forelimb |  |  |  | Hindlimb |  | |  | | | | | |
| --- | --- | --- | --- | --- | --- | --- | --- | --- | --- | --- | --- | --- | --- |
|  | ***Scapula*** | ***Humerus*** | ***Ulna*** | ***Radius*** | ***Femur*** | | ***Tibia*** | | | ***Fibula*** | |  |  |
| *E16.5 bone length (mm)* |  |  |  |  |  |  | |  | | |  |  |  |
| *Del/+*  *Del/Del* | 2.86 ± 0.09  2.77 ± 0.14 | 2.94 ± 0.09  2.83 ± 0.13 | 2.90 ± 0.10  2.87 ± 0.13 | 2.29 ± 0.08  2.24 ± 0.11 | 2.46 ± 0.08  2.37 ± 0.12 | 2.52 ± 0.10  2.42 ± 0.12 | | | 2.32 ± 0.10  2.25 ± 0.14 | | | |  |
| *E16.5 mineralized bone length (mm)* |  |  |  |  |  |  | | |  | | | |  |
| *Del/+*  *Del/Del* | 1.45 ± 0.07  1.43 ± 0.10 | 1.52 ± 0.08  1.44 ± 0.10 | 1.73 ± 0.08  1.70 ± 0.12 | 1.37 ± 0.06  1.36 ± 0.09 | 1.18 ± 0.06  1.14 ± 0.09 | 1.47 ± 0.07  1.43 ± 0.09 | | | 1.30 ± 0.06  1.29 ± 0.09 | | | |  |
|  |  |  |  |  |  |  | | |  | | | |  |
| *E18.5 bone length (mm)* |  |  |  |  |  |  | | |  | | | |  |
| *Del/+*  *Del/Del* | 3.97 ± 0.04  4.04 ± 0.02 | 4.13 ± 0.03  4.19 ± 0.02 | 4.16 ± 0.05  4.22 ± 0.06 | 3.23 ± 0.03  3.23 ± 0.03 | 3.61 ± 0.03  3.71 ± 0.02 | 3.78 ± 0.05  3.84 ± 0.03 | | | 3.50 ± 0.03  3.52 ± 0.04 | | | |  |
| *E18.5 mineralized bone length (mm)* |  |  |  |  |  |  | | |  | | | |  |
| *Del/+*  *Del/Del* | 2.38 ± 0.03  2.41 ± 0.03 | 2.50 ± 0.04  2.51 ± 0.03 | 2.87 ± 0.04  2.91 ± 0.03 | 2.27 ± 0.03  2.33 ± 0.03 | 2.03 ± 0.04  2.05 ± 0.03 | 2.49 ± 0.04  2.49 ± 0.05 | | | 2.31 ± 0.04  2.33 ± 0.04 | | | |  |
|  |  |  |  |  |  |  | | |  | | | |  |

Data are expressed as means with their standard errors. At E16.5, 18 control and 11 ninein-deleted embryos were analyzed. At E18.5, 20 control and 12 ninein-deleted embryos were analyzed. No difference in the total length and size of the mineralized area was observed in controls and ninein-deleted embryos.
